# Establishment of a free-mating, long-standing and highly productive laboratory colony of *Anopheles darlingi* from the Peruvian Amazon

**DOI:** 10.1186/s12936-015-0733-0

**Published:** 2015-05-30

**Authors:** Cuauhtémoc Villarreal-Treviño, Gissella M Vásquez, Victor M López-Sifuentes, Karin Escobedo-Vargas, Anibal Huayanay-Repetto, Yvonne-Marie Linton, Carmen Flores-Mendoza, Andrés G Lescano, Frederick M Stell

**Affiliations:** Centro Regional de Investigación en Salud Pública/Instituto Nacional de Salud Pública (CRISP/INSP), Tapachula, Chiapas Mexico; Department of Entomology, U.S. Naval Medical Research Unit No. 6 (NAMRU-6), Bellavista, Callao, Peru; Walter Reed Biosystematics Unit, Museum Support Center, Smithsonian Institution, Suitland, MD USA; Walter Reed Army Institute of Research, Silver Spring, MD USA; Department of Entomology, Smithsonian Institution, Washington, DC USA; Department of Parasitology, U.S. Naval Medical Research Unit No. 6 (NAMRU-6), Bellavista, Callao, Peru

**Keywords:** *Anopheles darlingi*, Colony establishment, Natural copulation induction, Larval rearing, Amazon basin, Peru, Malaria vector

## Abstract

**Background:**

*Anopheles darlingi* is the main malaria vector in the Amazon region and is among the most efficient malaria vectors worldwide. However, due to the lack of a well-established laboratory colony, key control-relevant aspects of the bionomics, behaviour, genetics, and vector-parasite relationships of *An. darlingi* remain unknown. Here, biological parameters that had been successful in initiating other *Anopheles* colonies were optimized and improved for *An. darlingi*, with the aim of establish a free-mating, stable, and highly productive laboratory colony.

**Methods:**

Wild *An. darlingi* adult females were field collected from Zungarococha, Loreto Department, Peru (03°49′32.40″S, 73°21′00.08″W), and taken to the NAMRU-6 Insectary in Iquitos where F_1_ offspring were produced and reared. Natural copulation was successfully induced in F_1_ adults under a thermoperiod of 30 ± 1 °C during the day and 25 ± 1 °C at night, and with a 30-min LED light stimulation period at dusk. Oviposition success was enhanced using egg-laying containers with a dark-coloured surface. Larval feeding regimes were standardized for optimal larval development. Optimized copulation induction methods were used to facilitate mating in *An. darlingi* until the F_10_ generation. No copulation induction assistance was needed in subsequent generations.

**Results:**

In 19 generations, the *An. darlingi* colony produced a total of 763,775 eggs; 441,124 larvae; 248,041 pupae; and 231,591 adults. A mean of 0.56 sexual encounters/female/cage (n = 36 cages) was recorded across the first ten generations (F_1_-F_10_). A mean insemination rate of 54.7 % (n = 5,907 females) ranging from 43.6 % (F_2_) to 66.6 % (F_10_) was recorded across nine generations (F_2_-F_10_). Free-mating was casually observed in the F_8_ generation, and subsequently confirmed in the F_9_ and F_10_ generations; comparable insemination rates and egg laying between stimulated (51.6 %, 12.9 eggs/female), and non-stimulated (52.3 %, 11.2 eggs/female) females were recorded. The time from egg to adult development ranged from 10 to 20 days. Moreover, the colony was relocated to a new laboratory within Iquitos in the F_14_ generation without any noted changes in its productivity. By March 2015, the *An. darlingi* colony has been successfully reared to the F_26_ generation.

**Conclusions:**

This constitutes the first report of a free-mating, highly productive, and long-standing *An. darlingi* laboratory colony established through natural copulation induction, which will support critical malaria research. This rearing methodology may be a transferable, cost-effective alternative to labour-intensive forced mating practices widely used in maintaining other *Anopheles* colonies.

**Electronic supplementary material:**

The online version of this article (doi:10.1186/s12936-015-0733-0) contains supplementary material, which is available to authorized users.

## Background

*Anopheles darlingi* is considered the most effective malaria vector in the Neotropical region [1, 2] and is responsible for most malaria transmission where it is found, including areas of high deforestation [3, 4]. This species efficiently transmits *Plasmodium falciparum*, *Plasmodium vivax* VK210, and *P. vivax* VK247 across Latin America [5–9], and its introduction to the northern Amazon has been linked to the considerable increase of *P. falciparum* cases in Peru in the 1990s [10, 11]. In Peru, *An. darlingi* is the dominant malaria vector in the Loreto Department, which is the most affected malaria endemic region in the country. *Anopheles darlingi* is present in both rural villages in the vicinity of Iquitos (15–20 km west-southwest) where it comprises 67–99 % of anophelines collected [12], and riverside camps along the Mazan river (40–50 km northeast of Iquitos) where 99 % of anophelines collected were identified as *An. darlingi* [13]. Interestingly, *An. darlingi* biting rates, and entomological inoculation rates (EIRs) differed considerably among these locations and are substantially higher at riverine sites [13], possibly linked to the observed malaria transmission heterogeneity in the Peruvian Amazon. Therefore, knowledge of the biology, behaviour, and vectorial capacity of this vector is crucial for understanding malaria transmission dynamics in the Amazon region, as well as for developing and implementing effective malaria vector-targeted control strategies. Laboratory colonies of *An. darlingi* reared up to the F9 generation have been reported before, producing less than 3,000 mosquitoes per generation [14, 15] and without description of long-term continuity. The lack of a highly-productive and well-established *An. darlingi* laboratory colony that can continuously provide large numbers of mosquitoes has limited these critical studies.

Previous reports regarding the establishment of a laboratory colony of *An. darlingi* [16–19] did not explain the main factors triggering sexual behavior and regulating colony productivity in this species. This historically limited replication of the methodology used and the establishment of new laboratory colonies. For example, low sexual activity resulting in limited oviposition in F_2_ adult females made establishment of a colony in São Paulo unsuccessful [19]. Recently, a method developed for *Anopheles pseudopunctipennis* was used for colonization of *An. darlingi* in the laboratory [20], yet very low densities of laboratory-reared adults across six generations (1,618 adults per generation on average) were reported. Moreover, critical characterization of sexual behaviour or parameters demonstrating adaptation of F_1_ to laboratory conditions and colony stability has not been reported to date [16–20]. This is an area with limited to no evidence. There is only one report from an *An. darlingi* mark-recapture study describing that sexual activity in field populations occurs in the evening shortly after the sunset and near human dwellings, yet no male swarms were observed despite the finding of inseminated recaptured females [20].

Thus, the main factors stimulating *An. darlingi* sexual behaviour and oviposition in the field and under laboratory conditions still remain unknown [14, 21]. Limited success of copulation induction in artificial environments such as an insectary, where adults are generally confined to rearing cages [19, 22, 23], remains the major blockage to colony establishment in *An. darlingi*. Forced copulation technique was explored in *An. darlingi* colony establishment, [24, 25]. Not only is this very difficult and time-consuming [14] but was also unsuccessful for *An. darlingi* with a very low percentage of F_1_ females (1 %) laying eggs (Escobedo and Huayanay, personal observation). The inability to induce copulation, in addition to the lack of an established method for mass larvae rearing, considerably impeded the establishment of a long-standing, highly productive *An. darlingi* laboratory colony.

Therefore, a natural copulation induction technique that has effectively worked for colonization of *An. pseudopunctipennis* and other anophelines including *An. darlingi* in Mexico although only until the F_9_ generation [14, 22], was evaluated, adapted and optimized. Also, conditions favoring egg-laying in gravid females were standardized and larvae rearing was improved by providing a diet that meets nutritional needs, has appropriate particle size, and is easily digestible [14, 22]. This combined approach has allowed to develop an effective method to establish and mass-rear *An. darlingi* under insectary conditions. The methodology used is described in all its complexity and detail to contribute to the development of other laboratory colonies across the Americas and for potential replication in other *Anopheles* species.

## Methods

### Study design

The F_1_ offspring of wild-caught *An. darlingi* females were used to evaluate and optimize techniques for natural copulation stimulation, oviposition induction, and larvae rearing. Sexual behaviour was examined by direct observation of mating across 10 generations (F_1_-F_10_), and video recording of adult behaviour during mating in F_1_ and F_14_ individuals. Successful mating was assessed by estimating the rate of inseminated females via spermatheca dissections across 10 generations (F_1_-F_10_). Free-mating was confirmed by comparing the rate of insemination and egg-laying between stimulated and non-stimulated F_9_ and F_10_ adults. Copulation induction was deemed unnecessary from the F_11_ generation onwards.

### *Anopheles darlingi* field collection and production of F_1_ adults

Adult females were collected in February 2013 (rainy season) in the community of Zungarococha (03º49′32.40″S, 73º21′00.08″W), 18 km southwest of Iquitos, Peru. Active transmission of *P. falciparum* and *P. vivax* historically occurs in this area [26], where *An. darlingi* has been incriminated as the main vector [12]. Adult females were captured hourly from 18:00 to 22:00 using protected human landing collections [25]. *Anopheles darlingi* females collected were placed in carton cups (250 ml) covered with nylon mesh, provided 10 % sugar solution and taken to the NAMRU-6 insectary in Iquitos. Here, cow, or chicken blood was offered through glass membrane feeders (3.8 cm outer diameter) to induce oviposition and obtain F_1_ adults following previously described procedures [21, 27]. Briefly, one wing of each blood-engorged female was cut and then females were placed individually in vials for egg laying. Eggs were inundated with water within 24 h and placed in plastic trays (26.5 × 16.5 cm). Larvae were transferred into plastic trays containing a mix of water from natural breeding sites and filtered water and fed a mixture of wheat flour and fish-meal with quantities increasing with larval development from 0.14 mg/larva (first instar) to 0.5 mg/larva (fourth instar). Pupae were transferred into plastic containers with water (200 pupae/container) and placed in screen cages for adult eclosion. F_1_ adults were used in natural copulation induction assays and for optimization of oviposition and larvae rearing. Morphological species identification of wild-caught and F_1_ adult *Anopheles darlingi* was conducted using keys for Neotropical *Anopheles* [28, 29]. Molecular verification of F_10_*An. darlingi* adults was confirmed using mitochondrial *cytochrome c oxidase I* (*COI*) gene sequences, following the standard protocol of the Mosquito Barcoding Initiative [30].

### Standardization of the natural copulation induction technique

The natural copulation induction technique developed for *An. pseudopunctipennis* [22] was initially evaluated against laboratory reared *An. darlingi* F_1_ adults obtained in February 2013. As the appropriate/critical space for copulation, sugar source finding, and mosquito density were unknown, two sizes of screened cages, medium (46 × 46 × 46 cm) and large (61 × 61 × 61 cm) were evaluated simultaneously following modifications on documented environmental conditions known to trigger natural copulation in *An. pseudopunctipennis* [22]. Briefly, 2–5 day old *An. darlingi* F_1_ males and females were placed in the two sizes of screened cages: 1,500 adults in the medium cage (1:1 females to males) and 2,400 adults in the large cage (1:1 females to males), and fed *ad libitum* with honey-water solution (10 %). Screened cages were kept in a room with controlled temperature that was maintained at 30 ± 1 ºC during the day (07:00 to 19:00) and at 25 ± 1 ºC during the night (19:00 to 07:00) and under a 12:12 light to dark photoperiod. Relative humidity was not controlled and ranged from 63 to 80 % following natural conditions in the city of Iquitos. F_1_ adult mosquitoes were exposed to a beam of a Light-Emitting Diode (LED) projected with a flashlight of 1.5 W placed 50 cm away from the cage. Three artificial 15-min periods of light exposure interjected by 5-min dark intervals were conducted at dusk for five consecutive days in a bid to increase sexual arousal and trigger natural copulation. Light exposure was carried out in complete silence to avoid disruption of mosquito sexual behaviour. Three days after the first copulation induction, F_1_ female mosquitoes were offered commercially purchased animal blood (either cow or chicken blood) via membrane feeders for 30–40 min. Blood meals were offered every two days for a period of 15 days from 1900 to 2000 h (recently induced females) and from 0900 to1030 h (ovipositing females); chicken blood was given twice during the first gonotrophic cycle (2–3 days) and either chicken or cow blood was provided at each subsequent feeding event. Fecundity (number of eggs laid per female) between females from cages that received only one type of blood versus those that received two types of blood was compared. In addition, wing length (mm) of a sample of females (10) per generation (F_1_–F_10_) was measured with a stereoscope (MEIJI EMZ-13, Saitama, Japan) at 30× magnification to examine changes in body size across generations, which could be linked to fecundity [31, 32]. These standardized copulation induction conditions were used to rear *An. darlingi* up to the F_10_ generation but using large cages only based on results from the cage-size evaluation.

### Assessment of *An. darlingi* sexual behavior and natural copulation under laboratory conditions

Direct observation of natural copulation in F_1_ mosquitoes was conducted in both medium and large cages during all three light stimulation periods for seven consecutive days. Mating pairs (copulations) were counted as mating pairs who encountered at flight and fell on the cage floor. Results are presented as total numbers of copulations/day/cage size as well as number of copulations/100 females, to account for the differences in total numbers of females placed in the medium and large cages. In subsequent generations (up to F_10_), the total number of copulations/female/cage was recorded but for only five days. Sexual behaviour of F_1_ and F_14_ males and females was recorded with a Nikon® D5100 video camera. The video was reviewed frame by frame in iPhoto’11 version 9.3 on a 1600 MHz iBook computer with an 11-in. video screen (Apple Computers, Cupertino, CA). Copulation time was recorded and sexual behaviour traits noted.

Insemination rates were estimated by dissecting the spermathecae of a representative sample of adult females collected from each cage across 9 generations (F_2_–F_10_), following the WHO standard methods [25]. Fallen females were collected daily for a period of 5–17 days, starting one day after the first day of copulation induction.

Apparent copulation in the absence of stimulation was first observed in the F_8_ generation. Direct observation of copulation in adults not stimulated by light and thermoperiod was conducted between 18:30 and 19:30 for the F_9_ and F_10_ generations. Free-mating behaviour was evaluated by comparing insemination rates per cage and egg-laying rates per female in those copulation-induced females versus non-stimulated controls. Insemination rates were determined as described above; egg-laying rates were estimated by dividing the total number of eggs laid per cage by the total number of females per cage.

### Optimization of *An. darlingi* oviposition and larvae rearing

Preliminary observations of oviposition preference of blood-fed F_1_ adult females were conducted in the NAMRU-6 Iquitos insectary, comparing light-coloured versus dark-coloured oviposition surfaces. Four white plastic trays (32 × 18 × 5 cm) and four additional identical trays covered with black plastic were filled with filtered water and placed into cages containing 2,000 F_1_ adults. Female attraction to each colored tray and oviposition rates were recorded. It was subsequently evaluated whether the addition of native aquatic plants (*Ceratopteris pteridoides*) commonly found in breeding sites would increase oviposition. Environmental conditions were identical for oviposition and natural copulation induction as eggs were collected in the same environment where cages with adults were kept. No honey solution was provided during the oviposition period (15 days). F_1_ eggs (c. 500) were transferred into larger plastic trays (37 × 25.5 × 5.5 cm) containing 1 - 1.5 L of filtered, dechlorinated water by directly pouring water from egg-collecting trays. These trays were kept at 28 ± 1 ºC, 66 ± 1 RH% and at a 12:12 light to dark photoperiod, conditions favourable for larval eclosion. In subsequent generations, eggs were collected on strips of filter paper (2 cm wide) lining tray edges to prevent egg damage.

Approximately 500 F_1_ first-instar larvae were reared in plastic trays (37 × 25.5 × 5.5 cm) until development of third-instar larvae. Third-instar larvae were divided into two trays so that a mean density of 250 larvae per tray was maintained until development of pupae. Larvae were fed commercially available rodent food (Laboratory Rodent Diet 5001, LabDiet®, St. Louis, MO) containing 23.0 % protein, 4.5 % fat, and 6.0 % crude fiber. Optimal feeding amount and frequency of dry rodent food fed were assessed and optimized for each larval instar as previously described [14, 22].

When larval mortality was higher than average, microscopic examination (40×) of water samples was performed from trays to determine the presence of bacteria, fungi and protists. Selective, and differential media (MacConkey agar, xylose lysine deoxycholate agar, and thiosulfate citrate bile salts sucrose agar) were used to differentiate bacteria.

Standardized oviposition and larvae rearing procedures have been used to rear *An. darlingi* up to the F_26_ generation. The colony had to be relocated to another NAMRU-6 facility in Iquitos while rearing the F_14_ generation, adaptation to laboratory rearing facilitated this process.

### Research ethics

Human landing collections were conducted following NAMRU-6′s security protocol, including explanation of risks, safety, healthcare, prophylaxis, and in case of disease, the appropriate care. Mosquito blood feeding using membrane feeders was conducted following the standard operating procedures of NAMRU-6.

### Statistical analyses

Reductions in larval mortality across generations were evaluated with Spearman’s Rho non-parametric correlation tests. Changes in egg, larvae and pupae productivity, and reductions in mortality associated with natural copulation were tested using Wilcoxon rank-sum (Mann–Whitney) non-parametric tests. Independent two sample *t*-tests assuming unequal variances (separate-variances *t*-tests) were performed for mean comparison of insemination rates and number of eggs laid by stimulated versus not-stimulated females; number of copulations/100 females in large versus medium cages; and number of eggs laid on black trays versus white trays. A Least Significant Difference (LSD) test was performed for mean comparison of female wing length. All analyses were conducted with Stata v13.0 (StataCorp, College Station, TX, 2013) except the LSD test that was conducted with SAS 9.4 (SAS Institute Inc., Cary, NC, 2002–2013). *P*-values < 0.05 were considered statistically significant.

## Results

### *Anopheles darlingi* colony establishment through natural copulation induction

The offspring (F_1_ individuals) of 878 wild-caught adult females from Zungarococha, Loreto served to establish an *An. darlingi* colony. Species identity of the laboratory colony was confirmed both by morphological analysis of wild-caught, F_1_ and F_10_ adults, and by DNA barcoding an F_10_ adult. The *COI* sequence data (658 bp, corresponding to the barcode region) was compared with expertly identified specimens in the databank of the Mosquito Barcoding Initiative (Barcode of Life Database, BOLD), and those available in GenBank. The colony sequence (GenBank accession number KP193458) showed 99.6–100 % identity with other *An. darlingi* sequences from Peru, Bolivia, and Brazil in the Barcode of Life Database (BOLD) [33]. Comparison with published GenBank entries revealed highest identity with JF923693 from Acrelandia, Brazil, (99.34 %) and 99.2 % and 98.8 % identity with GQ918272 and GQ918273, the complete mitochondrial genome from the northern and southern lineages of *An. darlingi,* respectively [34].

**Fig. 1:**
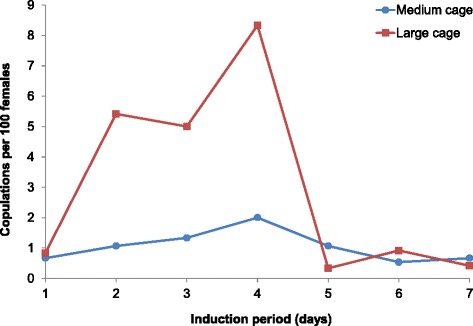
Copulation induction period and number of natural copulations observed per 100 *An. darlingi* F_1_ adult females kept in medium (46 × 46 × 46 cm) and large (61 61 × 61 cm) cages under laboratory conditions in the NAMRU-6 insectary in Iquitos, Peru

During first attempts of applying and optimizing thermoperiod and light exposure regimes used for *An. pseudopunctipennis* [22] in February 2013, it was observed, and digitally recorded for the first time *An. darlingi* natural copulation under laboratory conditions. This significant milestone is publicly available in Additional file 1A, and documents the sexual behavior of *An. darlingi* for the first time. In this first experiment, copulation induction was tested in medium and large screened cages. In both cage sizes, copulation increased from day 1 to day 4, and then decreased as shown by the daily number of natural copulations (Table 1) and the number of copulations per 100 females (Fig. 1). In the medium-sized cage, the highest number of natural copulations was recorded on day 4 (15 total: two per 100 females), and the lowest number on day 6 (four total: 0.5 per 100 females). Copulation was substantially higher in the large cage on days 2–4 (60–100 total: 5–8 per 100 females). The number of copulations per 100 females (mean ± standard deviation) on days 2–4 was significantly higher in the large cage (6.3 ± 1.8) than in the medium cage (1.5 ± 0.5) (*t*-test: *p* = 0.0238). Therefore, large cages with a density of approximately 2,500 adult mosquitoes were used since to provide the best conditions for natural copulation in subsequent generations. Copulation induction was conducted only for five days up to the F_10_ generation when free-mating was confirmed.

**Table 1:** Number of natural copulations recorded for different thermoperiod/light induction periods for An. darlingi enclosed in cages of two different sizes (medium = 46 × 46 ×46 cm; large = 61 × 61 × 61 cm) and with different adult densities

Period of natural copulation induction (days)	Medium cage	Large cage
	1,500 adults	2,400 adults
	(1:1 ♂:♀)	(1:1 ♂:♀)
1	5	10
2	8	65
3	10	60
4	15	100
5	8	4
6	4	11
7	5	5
Total number of copulations	55	255

F_1_ adults tested were 2–3 days old. Observations were conducted in the NAMRU-6 insectary in Iquitos, Peru

**Fig. 2:**
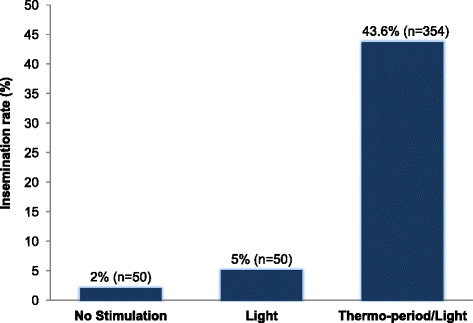
*Anopheles darlingi* insemination rates (%) recorded for adult females that received i) no copulation stimulation, ii) copulation stimulation using light only, and iii) copulation stimulation using thermoperiod and light. Copulation data without stimulation, and light only copulation stimulation data were taken from [22]. Insemination rates estimated under no stimulation and light only conditions were recorded in F_1_ adult females, and under thermoperiod/light in F_2_ adult females. Adult females were reared in the NAMRU-6 insectary in Iquitos, Peru

The percent insemination rate recorded in *An. darlingi* F_2_ adult females was 43.6 ± 5.4 %. This insemination rate was higher when compared to those previously recorded in F_1_ adult females reared at the NAMRU-6 Insectary [21] that had not been stimulated, 2 ± 2.8 %, or stimulated with light only, 5 ± 1.4 % (Fig. 2). Copulation induction using a combination of light and thermoperiod as stimuli was more effective in triggering adult copulation as evidenced by the higher insemination rate recorded relative to those recorded with no stimulus or stimulated by light only.

### Sexual behavior, copulation, and insemination rates in colonized *Anopheles darlingi*

General aspects of the *An. darlingi* sexual behaviour were examined frame by frame in a 17-min video recorded during copulation induction of F_1_ adults (Additional file 1A). Forty-five natural copulations at the rate of approximately 2.6 copulations per minute were recorded. Males and females mated in midair and copulation lasted on average 1.9 (± 2.2) seconds, ranging from 1 to 10 s. In nearly all observed copulations (97.7 %), the mating pair fell on the cage floor and 84.1 % (37 of 44), where they remained for less than a second, before separating, and flying away. The remaining seven couplets stayed together for over a second. After copulation ended, males, and females separated from each other, and females immediately flew away. Only a fraction of males (51.1 %) that had separated from females stayed on the cage floor for 6.1 (± 7.8) seconds on average in apparent rest or recovery, and then flew away. Flight height above cage floor, wing flapping frequency, copulation in midair, length of copulation, in F_14_ adults was similar to that of F_1_ adults, but copulation rates were 7.7 times higher, at c. 20 copulations per minute (Additional file 1B).

**Fig. 3:**
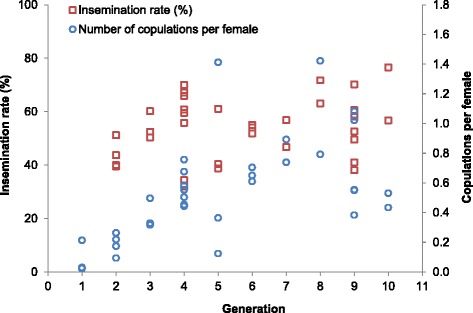
*Anopheles darlingi* copulations per female and insemination rates (%) recorded across 10 generations (F_1_–F_10_) reared in the NAMRU-6 insectary in Iquitos, Peru

The mean number of copulations/female/cage (n = 36) increased significantly across the first ten generations (Spearman’s rho = 0.66; *p* < 0.0001) (Fig. 3). The lowest mean number of copulations/female/cage was recorded for the F_1_ generation (0.1 ± 0.1) and the highest for the F_8_ generation (1.1 ± 0. 5). The mean insemination rate (%) recorded per cage (n = 34; 45–367 females sampled per cage) increased at a marginal significant rate (Spearman’s rho = 0.26; *p* < 0.1340) across the first nine generations (F_2_–F_9_) (Fig. 3). The lowest mean insemination rate was recorded for the F_2_ generation (43.6 ± 5.4), while the highest for the F_8_ generation (67.4 ± 6.1). Mean insemination rates have reached > 50 % since the F_6_ generation.

In females from the first ten generations, egg laying per female did not differ between females offered one type of animal blood (17.2 ± 14.9 eggs/female) and those given two choices of animal blood (26.5 ± 22.7 eggs/female) (*t*-test: *p* = 0.0808). However, significant differences were detected in wing length of females from the F_1_-F_10_ generations and the Zungarococha field population (*F* = 6.40; *p* < 0.0001), with the smallest wing length (2.70 ± 0.11 mm) recorded in the F_2_ generation and the largest (3.04 ± 0.13 mm) for wild-caught females. F_9_, F_10,_ and wild-caught females had larger wings than females from generations F_1_–F_8_ (LSD *p* < 0.05), suggesting that female fecundity in the first eight generations could have been lower than that of field populations, yet comparable in later generations [31, 32].

The occurrence of free-mating was first recorded in the F_8_ generation upon occasional observation of copulation in the absence of stimulation, therefore direct observation of free mating and comparison of insemination rate and egg laying between stimulated and non-stimulated females were conducted in the F_9_ and F_10_ generations. Moreover, there were no significant differences in the mean insemination rates per cage recorded for stimulated (51.6 ± 12.7 %) and non-stimulated (52.3 ± 2.9 %) females from the F_9_ and F_10_ generations (*t*-test: *p* = 0.4563). Likewise, the numbers of eggs laid per stimulated (12.9 ± 4.4) and non-stimulated (11.2 ± 7.3) females did not significantly differ (*t*-test: *p* = 0.2896). These results confirmed that this *An. darlingi* colony was self-mating, therefore, copulation induction procedures were ceased from the F_11_ generation. The colony has continued through the F_26_ generation with an increase in egg and adult production.

### Productivity and continuity of the *Anopheles darlingi* laboratory colony

Oviposition preference experiments showed that the mean number of eggs laid on trays covered with black plastic (84.5 ± 24.4) was significantly higher than those laid on white trays (16 ± 2.2) (*t*-test: *p* = 0.0056), suggesting that *An. darlingi* females preferred to lay their eggs on darker strata. It was determined that 20 mg per tray given at a frequency of 1, 2, 3, and 4 times per day for first, second, third, and fourth instars respectively, was optimal for larval development and also reduced water contamination due to bacterial growth on excess food. The estimated number of larvae per tray was 500 for first and second instars, and 250 for third, and fourth instars. These oviposition conditions and larval feeding regimes have been used for continuous rearing of colonized *An. darlingi.*

The *An. darlingi* colony has been maintained for 26 generations (February 2013 – March 2015), indicating successful adaptation to insectary conditions. An additional file shows the *An. darlingi* immature and adult rearing rooms in the NAMRU-6 Insectary in Iquitos (see Additional file 2). Data recorded across 19 successive generations (F_1_–F_19_) strongly demonstrate the overall steadiness and increasing productivity of this free-mating colony as shown by the production of a total of 763,775 eggs; 441,124 larvae; 248,041 pupae; and 231,591 adults (data collected from February 2013 to June 2014) (Table 2). It is possible that the colony’s free-mating nature and relative stability facilitated its re-establishment in this new facility where its rearing was continued up to the F_26_ generation to date.

**Table 2:** Total numbers of eggs, larvae, pupae, and adults; and percentage of unhatched eggs, larval, and pupal mortality of An. darlingi reared for 19 generations (F_1_–F_19_) in the NAMRU-6 insectary in Iquitos, Peru

Generation	No. of cages	Copulation induction period (days)	Total eggs	Unhatched eggs (%)	Total larvae	Larva-pupa mortality (%)	Total pupae	Pupa-adult mortality (%)	Total adults^a^
1	4	5	5,000	4.0	4,800	4.2	4,600	8.0	4,230
2	4	5	10,000	19.4	8,063	0.9	7,993	5.0	7,593
3	3	5	10,590	61.3	4,095	5.8	3,859	4.0	3,704
4	8	5	29,380	48.9	15,000	44.5^b^	8,327	1.3	8,216
5	3	5	35,419	22.0	27,616	93.3^c^	1,845	34.0^c^	1,218
6	3	5	14,560	55.0	6,550	51.4	3,184	6.2	2,987
7	2	5	18,790	33.5	12,500	47.6	6,544	8.3	6,000
8	2	5	4,950	29.3	3,500	54.7	1,586	6.4	1,485
9	7	0/5^d^	24,000	47.9	12,500	35.4	8,073	7.6	7,460
10	10	0/3^e^	43,810	44.1	24,500	11.8	21,619	6.5	20,205
11	7	0	46,600	57.1	20,000	13.3	17,339	4.9	16,485
12	6	0	45,450	45.0	25,000	4.8	23,790	8.9	21,664
13	5	0	49,130	49.1	25,000	40.1	14,976	9.0	13,629
14	8	0	61,750	51.4	30,000	86.1^c^	4,183	21.4^c^	3,289
15	6	0	54,000	55.6	24,000	46.0	12,951	8.9	11,801
16	9	0	116,465	59.6	47,000	54.7	21,272	7.0	19,773
17	6	0	57,100	29.9	40,000	82.2^c^	7,130	11.0	6,345
18	6	0	53,880	33.2	36,000	67.2	11,820	6.5	11,057
19	7	0	82,901	9.5	75,000	10.7	66,950	3.7	64,450
TOTAL			763,775		441,124		248,041		231,591
MEAN (SD)			40,199 (28,594)	39.8 (17.1)	23,217 (17,756)	39.7 (29.6)	13,055 (14,765)	8.9 (7.3)	12,189 (14,202)
95 % CI			26,417–53,981	31.5–48.0	14,659–31,775	25.5–54.0	5,938–20,171	5.4–12.4	5,344–19,034

^a^Mean sex ratio (male:female) across generations is 1:1

^b^Presence of *Enterobacter* sp*.* and *Serratia marcescens*

^c^Presence of fungus and protists

^d^Copulation induction performed for 5 days only in 4 cages

^e^Copulation induction performed for 3 days only in 2 cages

**Fig. 4:**
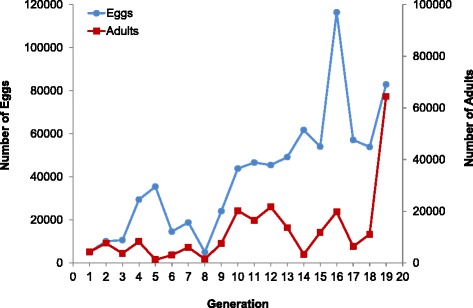
Production of *An. darlingi* eggs and adults across 19 generations (F_1_–F_12_) reared in the NAMRU-6 insectary in Iquitos, Peru

**Fig. 5:**
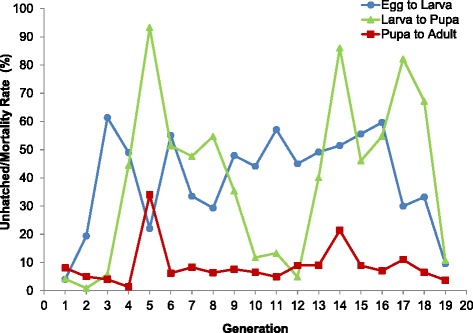
*Anopheles darlingi* unhatched egg rates (%), and larval, and pupal mortality (%) across 19 generations (F_1_–F_12_) reared in the NAMRU-6 insectary in Iquitos, Peru. Free-mating was recorded from generation F_9_

During this laboratory adaptation process, total numbers of eggs produced increased across generations (Fig. 4), although the percent of unhatched eggs varied (Fig. 5). The total number of eggs laid per generation ranged from 4,950 to 116,465, and the percent of unhatched eggs ranged from 4.0 % to 61.3 % (Table 2). The rate of unhatched eggs was above 60 % in a few generations (F_3,_ F_6_, F_11,_ and F_16_), but below 50 % for all other generations (Fig. 5). F_13_ eggs were checked under the micro stereoscope five days after placement in hatching trays to observe whether they had opened opercula and verify egg fertilization. Observations made on F_13_ eggs with a closed operculum (unhatched eggs) indicated that these were mainly unfertilized eggs rather than unviable/damaged fertilized eggs, which may be in part linked to insemination rates recorded (54.7 ± 7.9 %, mean across nine generations).

The total number of larvae produced per generation ranged from 3,500 to 75,000 with a mean larva-pupa mortality of 39.7 ± 29.6 % (Table 2). Larval mortality was less than 10 % in the first three generations, but increased in subsequent generations with peaks (>80 %) recorded for F_5_, F_14,_ and F_17_ larvae (Fig. 5). Larval mortality was most likely associated with bacterial infections (*Enterobacter* sp., *Serratia marcescens*, *Pseudomonas* sp.) as observed in F_4_ larvae, and / or water contamination by fungi and protozoa as observed when rearing F_5_, F_14,_ and F_17_ larvae. In addition, rodent food availability was limited for F_5_ larvae, and was temporarily replaced by spirulina (200 mg, first and second instars), and Koi food (120–300 mg, third, and fourth instars). Rodent food was available again for the F_6_ generation, which helped reduce larval mortality in this generation. A significant reduction in larval mortality (*p* = 0.0020) was recorded after the contamination/diet issues in the F_5_ generation but only up to the F_12_ generation where larval mortality rates were comparable to the lowest rates recorded in the first three generations (Fig. 5). However, water contamination again led to high mortality in F_14_ and F_17_ immatures. To reduce water contamination issues, larvae rearing trays were covered with plastic screens to avoid exposure to pathogens commonly found in the environment.

Pupae production ranged from 1,586 to 66,950 with a mean pupal mortality of 8.9 % (± 7.3 %) that has been under 12 % in all generations except for the F_5_ (34.0 %) and F_14_ (21.4 %), due to presence of fungus and protists (Table 2, Fig. 5). The total number of adults produced per generation also varied with the lowest (1,218) in the F_5_ and the highest (64,450) in the F_19_ (Table 2, Fig. 4).

Overall, egg, and adult productivity increased considerably after the F_8_ generation (*p* = 0.002 and *p* = 0.0179, respectively) (Fig. 4). Interestingly, increasing egg and adult numbers were recorded after completely ceasing copulation induction methods from the F_11_ generation (free-mating).

Based on observations made across 19 generations, *An. darlingi* egg to adult development under laboratory conditions was on average 13.7 ± 2.3 days and ranged from 10 (F_10_) to 20 (F_5_) days. A longer egg to adult development ranging from 12 to 29 days was recorded for *An. darlingi* F_1_ reared under laboratory conditions [14], yet the average recorded for the colony lies within this range. Egg to larval development required two days. First instar larva to pupal development ranged from 6 to 16 days and pupal to adult development required two days. An additional file shows photos of the different stages of *An. darlingi* [see Additional file 3].

## Discussion

Herein it is reported the successful establishment of the first free-mating, highly productive, and long-lasting *An. darlingi* colony by: a) inducing natural copulation through a combination of optimal thermo-period and light stimulation; and b) optimizing egg oviposition and larval rearing parameters. Despite the relevance of *An. darlingi* for malaria transmission in the Amazonian region [10, 11], many aspects of its physiology, behavior, ecology, genetics, and interaction with *Plasmodium* spp. are poorly understood due to the lack of a laboratory colony that can serve as a source of large numbers of individuals [35, 36]. This large and highly productive colony is a unique, valuable resource for developing, and evaluating effective vector-based strategies against malaria transmission in the Amazon. Extensive methodological details are provided to the scientific community to encourage the reproduction of the results, establishment of additional colonies, and extrapolation of the described methods to critical *Anopheles* vectors in the Amazon and in other regions of the world.

Historically the establishment of a laboratory colony of *An. darlingi* proved difficult, and this is the first report of a free-mating, autonomous colony of this important vector species. A recent study showed successful colonization of *An. darlingi* and *P. vivax* infection of colonized mosquitoes from F_3_ to F_6_ generations [15], yet no evidence of free-mating or a description of sexual behaviour was reported. Low sexual activity observed in adults confined to screened cages under insectary conditions has been the main cited obstacle in establishing an *An. darlingi* colony [23]. In this study it is shown that natural copulation occurs consistently and effectively in the laboratory with four-fold higher copulation rates in larger cages (61 × 61 × 61 cm). Additionally, external diurnal stimuli, was initially required to trigger sexual behaviour. This involved increased temperatures during the day and lower temperatures during twilight, in addition to exposure to a dim light beam at dusk, as to resemble field conditions. Light beams at dusk may resemble natural sunbeams decreasing at this time of the day, thus cueing mosquitoes that the sun is setting and potentially triggering their mating behaviour. These conditions sparked sexual activity in cages immediately after turning the lights off. In previous studies of *An. darlingi* reproductive behaviour in the NAMRU-6 Insectary, a very small percentage of caged adult females were inseminated in the absence of any type of stimulus or when applying a light beam for 30-min during twilight [14]. In this study, a specific thermoperiod regime coupled to exposure to a LED beam resulted in a considerably higher mean insemination rate (55 %). This is in line with field studies where virgin females were released in the field at sunset, and when recaptured two hours later, 60 % had been inseminated [20]. Sexual activity increased across generations from 2.6 (F_1_) to 40 (F_14_) matings per minute. Light stimulation was not needed after the F_10_ generation indicating the development and selection of a stenogamic colony.

It was also found that *An. darlingi* copulation rates were higher in 2 to 8 day-old mosquitoes, suggesting that the sexual stimulation method should be applied to that specific age range. Older females may not respond as effectively. Observations in colonized *An. darlingi* agree with those recorded in *An. gambiae*, for which the optimal age for male swarming in the field and female copulation success in the laboratory was found to be 4–8 days [37]. In the field, *An. darlingi* males probably fly at low altitudes, close to the vegetation, and remaining near breeding sites waiting to copulate, and even some may travel to food sources following females [20]. In this study, and in line with the hypothesized low flying altitude in the field, males were observed flying 5–40 cm above the cage floor, and forming a pseudo-swarm, and apparently attracting females. Adults copulated while flying and fell to the cage floor with most mating pairs separating in less than second. All of these behaviors are similar to that of Neotropical *Anopheles* reared under laboratory conditions [22, 38]. Also, some apparent male competition for females was observed in this study, with two males attempting copulation simultaneously with the same female and all three falling together on the cage floor, which could have interfere with copulation. Further examination is needed to assess if this behaviour had an effect on copulation success.

Previously, an *An. darlingi* population from Lacandon Forest in Chiapas, Mexico was colonized by natural copulation induction and reared for nine generations. However, the colony collapsed after females stopped ovipositing in white containers placed inside cages [14]. In this study, this problem was prevented by using highly-preferred dark oviposition trays that perhaps better resemble natural oviposition sites. Native aquatic plants in oviposition trays served as resting places for ovipositing adults and larvae, but were removed from the rearing protocol as they appeared to be a water contamination source.

In addition to optimizing oviposition, an optimal larval feeding regime was established using commercially available rodent food. This food appears to meet the nutritional needs of the immature *An. darlingi* larvae in the laboratory, and has been tested in other *Anopheles* spp. effectively [22]. Other commercial animal foods (fish, dog, or monkey) appear to be not as suitable to rear *An. darlingi* larvae possibly because their high fat content could lead to the formation of an oily/greasy layer on top of the water, which seems to kill larvae and promote the development of pathogenic microorganisms.

The establishment of autonomous neotropical anopheline colonies under insectary conditions has had variable success. Some anopheline species are considered relatively easy to colonize, such as *Anopheles albimanus* that does not require copulation induction [38]. In contrast, *An. pseudopunctipennis* requires artificial induction of mating [22]. In addition, the number of generations needed to develop an autonomous colony also varies by species and population origin. For example, the Tapachula, and Abasolo Mexican laboratory strains of *An. pseudopunctipennis* required 5 and 12 generations to select a free-mating population, respectively [22]. A stenogamous colony of *Anopheles albitarsis* from Brazil was obtained in six generations [39] while free-mating in *Anopheles aquasalis* was recorded in the F_2_ generation [40]. Finally, the previously attempted Mexican *An. darlingi* colony collapsed at the F_9_ generation before becoming autonomous [14], yet the Peruvian *An. darlingi* colony became autonomous after nine generations. Therefore, it is suggested that free-mating in colonized *An. darlingi* can only be demonstrated after rearing at least nine generations.

The fact that this study reports the first establishment of a stenogamic laboratory colony of *An. darlingi* despite many previous attempts [14, 16, 19] proves that colonizing and rearing this species is very challenging. The *An. darlingi* colonization approach used involved three critical steps: 1) employment of a very rigorous yet replicable procedure to trigger sexual behavior leading to successful insemination, 2) optimization of proper conditions for consistent oviposition, and 3) standardization of an adequate immature feeding and rearing regime. This approach produced a practical method to establish *An. darlingi* colonies and represents an alternative to the time-consuming and unpractical forced-mating method. The overall approach can be easily adapted and replicated for the establishment of other *An. darlingi* colonies, and could be useful to establish colonies of other critical malaria vectors worldwide.

Unlimited access to *An. darlingi* colony material will allow us to examine and understand unknown biological aspects of this important malaria vector including longevity, gonotrophic cycle length, oviposition behaviour, fecundity and so forth, as well as behavioural, and genetic traits that can be exploited for the development of novel and effective vector control approaches. It is also important to better understand male traits that determine copulation success, such as body size, nutritional status, and any associated genetic components, to facilitate future release of genetically altered males refractory to *Plasmodium* spp. Additionally, the interaction of *An. darlingi* with *Plasmodium* spp. parasites under controlled laboratory conditions and the performance of novel drug therapies targeting parasite development in the mosquito can now be better examined. Preliminary *P. vivax* infection studies via membrane feeding assays (4 successful experimental infections) conducted in colonized *An. darlingi* adult females from the F_19_–F_21_ generations have shown that these colonized mosquitoes are susceptible to *P. vivax* infection, with number of oocysts per mosquito ranging from 2 to 197 (mean ± SD = 38.6 ± 41.5), and number of sporozoites per mosquito ranging from 152 to 61,867 (mean ± SD = 8,141.4 ± 9,489.7). These values are comparable with those reported for colonized *An. darlingi* (F_1_–F_6_) experimentally infected with *P. vivax,* with oocyst number per mosquito ranging from 1 to 57 and sporozoite number per mosquito ranging from 150 to 7,380 [15]. These preliminary results will be confirmed by additional *P. vivax* experimental infections of colonized *An. darlingi* from the F_22_–F_26_ generations. Overall, information gathered from physiological, behavioural, and experimental infection studies conducted in colonized *An. darlingi* will be useful in developing strategies for comprehensive, integrated malaria vector control.

## Conclusions

Herein it is provided the first report of a well-established, highly productive, stenogamous colony of *An. darlingi*, having produced a total of 231,591 adults in 19 generations. Mating behavior of *An. darlingi* under laboratory conditions is described for the first time. Methods developed here may well prove useful to start additional *An. darlingi* colonies and in the establishment of other difficult malaria vector species. Having an established *An. darlingi* laboratory colony represents an invaluable resource for critical malaria research in the Amazon region.

## Additional files

Additional file 1:
**Video of natural copulation under laboratory conditions in** ***An. darlingi*** **F**_**1**_ **adults (A) and F**_**14**_ **adults (B).**
Additional file 2:
***Anopheles darlingi*** **rearing rooms in the NAMRU-6 insectary in Iquitos.**
Additional file 3:
**Developmental stages of** ***An. darlingi.***
