# Supplementary figures and images for: Establishment of a free-mating, long-standing and highly productive laboratory colony of Anopheles darlingi from the Peruvian Amazon
Source: Malar J. 2015 May 30;14:227. doi: 10.1186/s12936-015-0733-0 (PMC4465318; doi:10.1186/s12936-015-0733-0)

## A) Immature rearing room

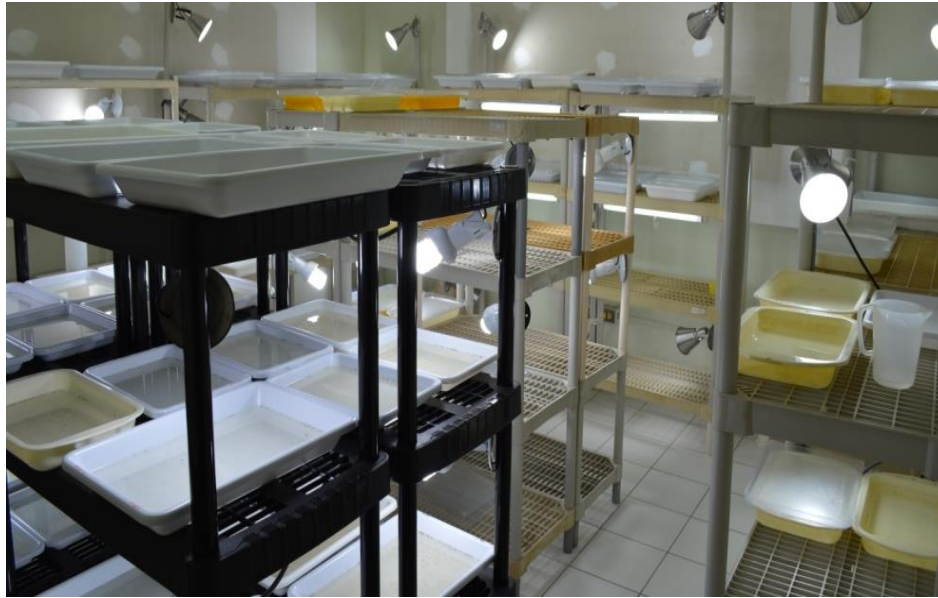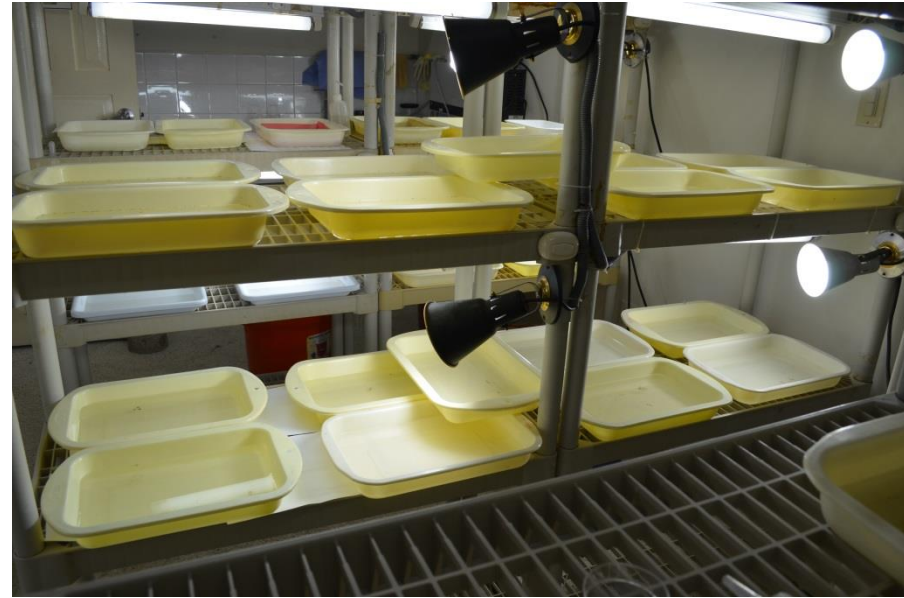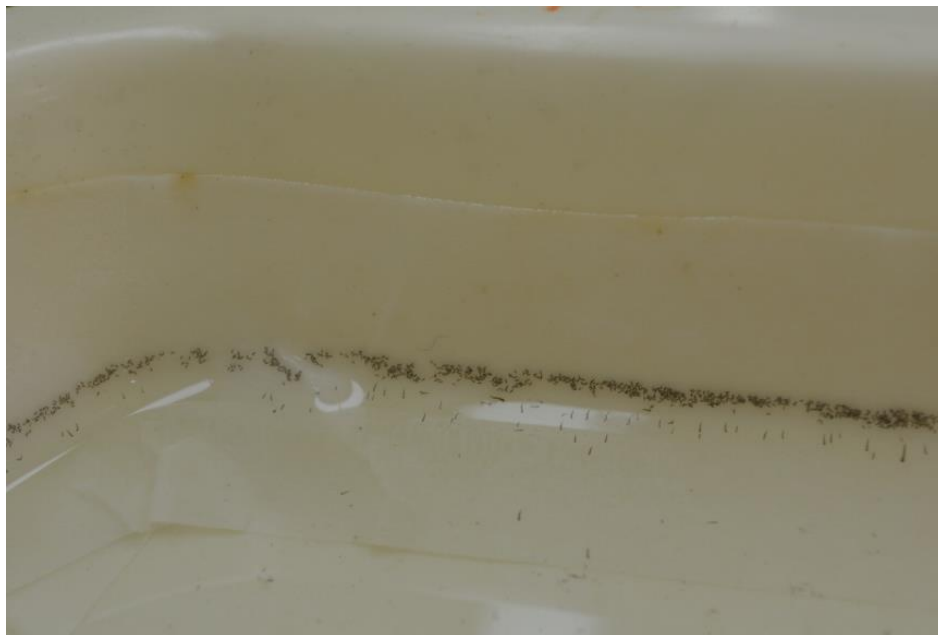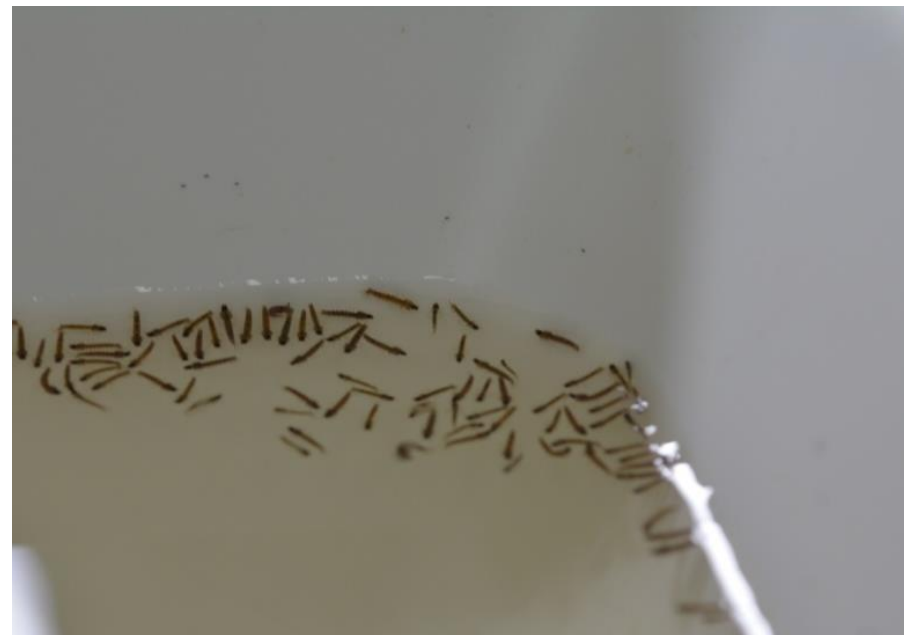

## B) Adult rearing room

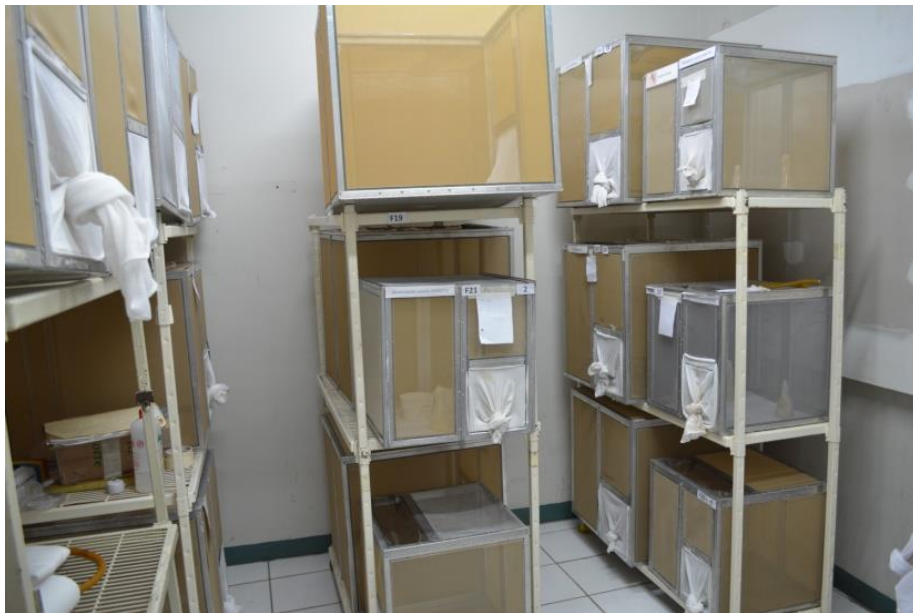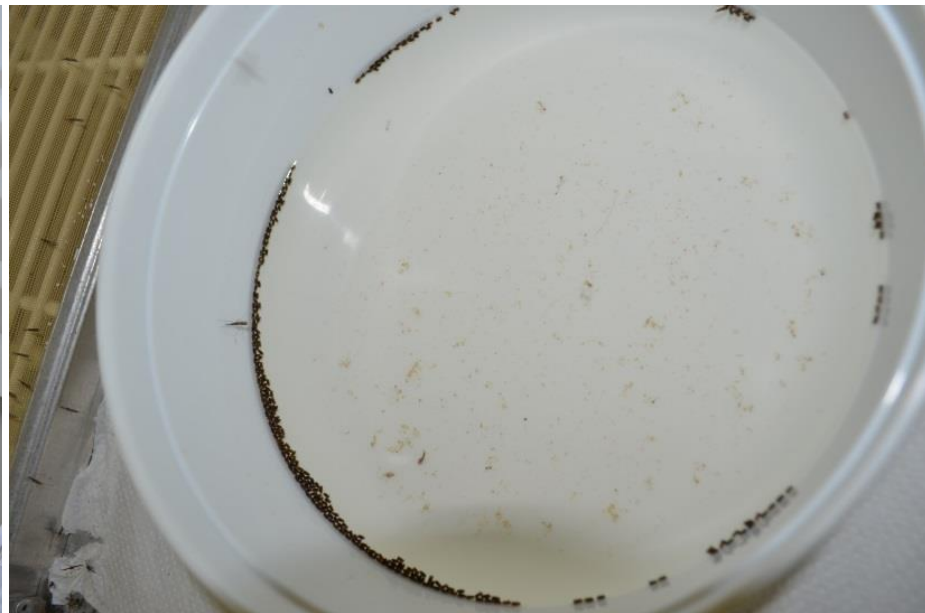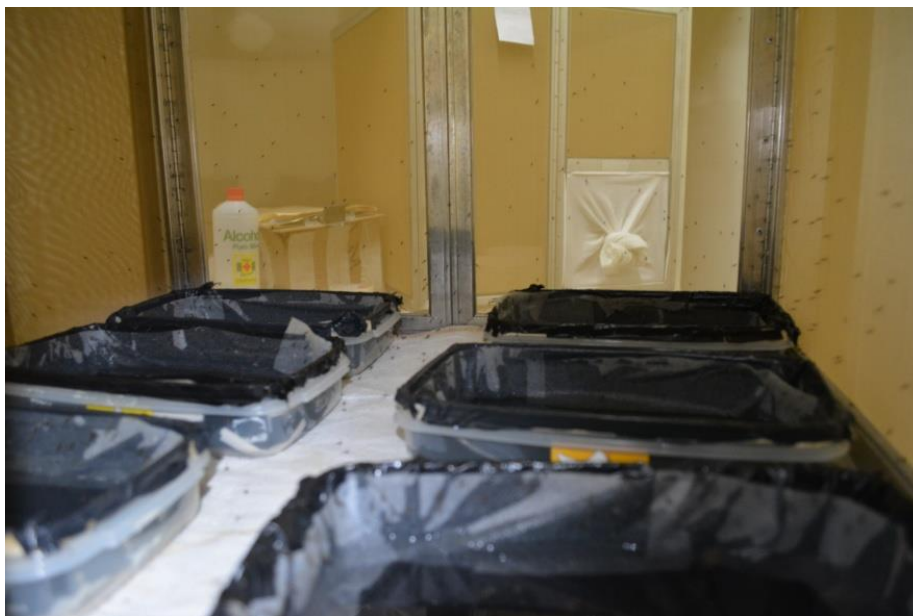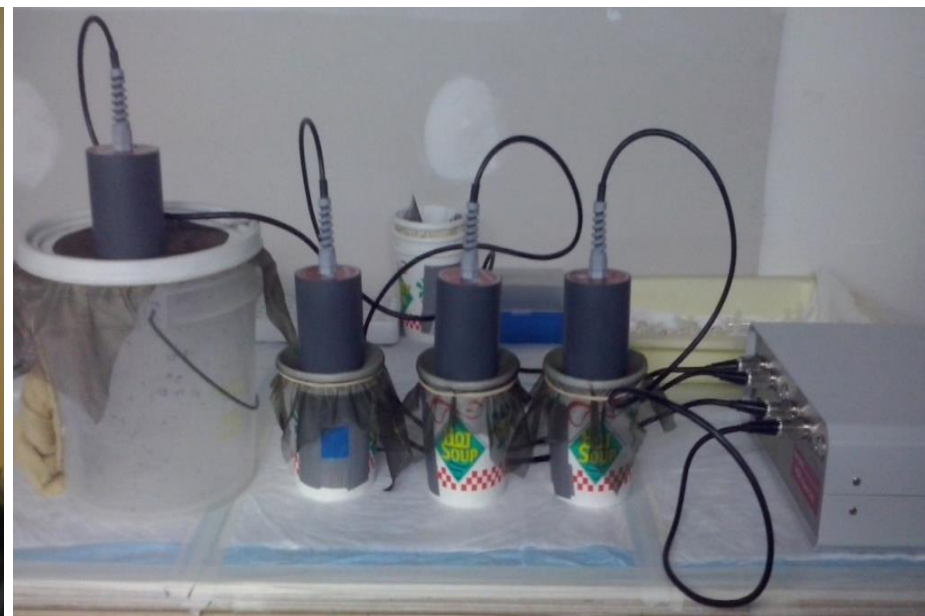

Supplement: Additional file 2: — Anopheles darlingi rearing rooms in the NAMRU-6 insectary in Iquitos. [file 12936_2015_733_MOESM2_ESM.pdf]
