# Supplementary material for: Establishment of a free-mating, long-standing and highly productive laboratory colony of Anopheles darlingi from the Peruvian Amazon
Source: Malar J. 2015 May 30;14:227. doi: 10.1186/s12936-015-0733-0 (PMC4465318; doi:10.1186/s12936-015-0733-0)
Supplement: Additional file 3: — Developmental stages of An. darlingi. [file 12936_2015_733_MOESM3_ESM.pdf]

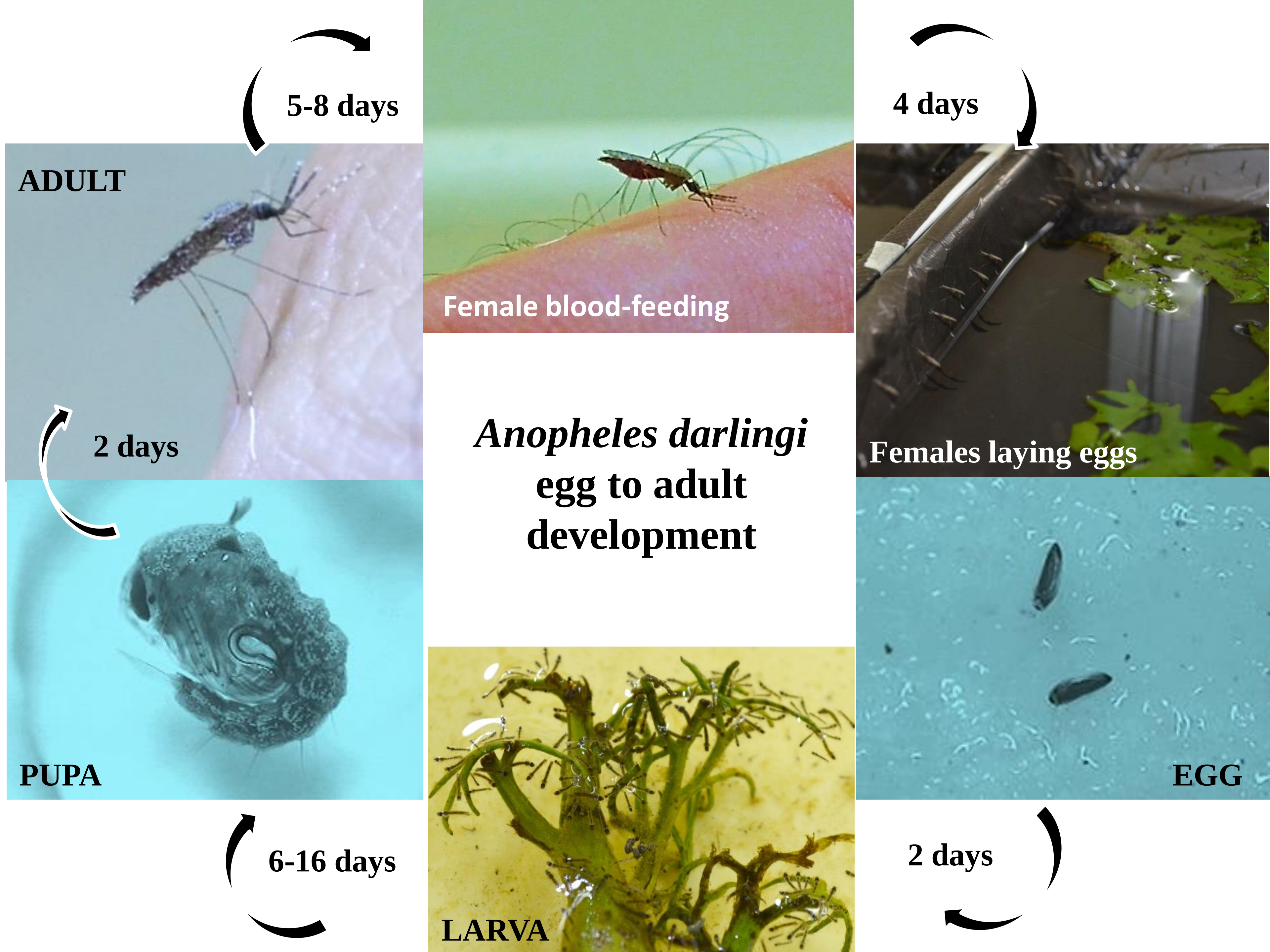
5-8 days
Female blood-feeding
4 days
ADULT
Females laying eggs
2 days
Anopheles darlingi
egg to adult
development
PUPA
EGG
6-16 days
2 days
LARVA
